# Governing Synthetic Biology: A Co-Evolutionary Framework for Sustainable Innovation

**DOI:** 10.4014/jmb.2508.08001

**Published:** 2025-11-26

**Authors:** Hyeonsu Kim, Jiyeon Lee, Yejin Cho, Haseong Kim, Heoung-yeol Kim, Bong Hyun Sung

**Affiliations:** 1 National Biotechnology Policy Research Center, Korea Research Institute of Bioscience and Biotechnology (KRIBB), Daejeon 34141, Republic of Korea; 2 Graduate School of National Public Policy, Chungnam National University, Daejeon 34134, Republic of Korea; 3 Synthetic Biology Research Center, Korea Research Institute of Bioscience and Biotechnology (KRIBB), Daejeon 34141, Republic of Korea; 4 Department of Biosystems and Bioengineering, Korea University of Science and Technology (UST), Daejeon 34113, Republic of Korea

**Keywords:** Synthetic biology, biomanufacturing, regulatory governance, societal acceptance, anticipatory governance

## Abstract

Synthetic biology has rapidly evolved from laboratory-based research to a core technology driving biomanufacturing, industrial innovation, and the global bioeconomy. Technological advancements such as next-generation sequencing, DNA synthesis, novel genome editing, and biofoundry automation are accelerating the industrial expansion and application of synthetic biology. However, despite these breakthroughs, current regulatory frameworks and societal acceptance are not in pace with technological development, creating significant barriers to the sustainable advancement of synthetic biology. This review offers a chronological analysis of synthetic biology development, examines regulatory and policy challenges, and proposes a 'co-evolutionary' model based on the 'Framework for Anticipatory Governance of Emerging Technologies' given by the Organization for Economic Co-operation and Development (OECD). Specifically, we highlight five key elements, research and development, strategic intelligence, societal trust formation, agile regulation, and international cooperation, as the foundation for mutual evolution of technology and institutional frameworks. Transforming science and technology into societal value requires essential policy support, which is a critical task for not only policymakers, but also scientists. Scientists must consider the societal context and institutional conditions in which their research operates, thereby ensuring the accountability and sustainability of science and technology. The future of synthetic biology requires a parallel approach that integrates scientific and technological advances with societal science perspectives, underpinned by collaborative governance among scientists, policymakers, and civil society.

## Introduction

While the 20th century was characterized by chemical-based industrialization, the 21^st^ century represents the era where the industrialization of biology has begun [1]. In line with these contemporary trends, the importance of synthetic biology is becoming increasingly prominent. The term 'synthetic biology' was first used in Stephane Leduc's publication in 1912, but the concept of synthesizing living organisms had been attempted even before Leduc [2, 3]. Jacques Loeb (1899), who became a Nobel Prize candidate (1901) for his discovery of 'artificial parthenogenesis,' successfully achieved sea urchin embryonic development through chemical treatment and claimed that living organisms could be artificially produced in the laboratory [4]. John Butler Burke (1905) generated half-living forms using radium-induced growths called “radiobes”, sparking a debate in the history of science about the origin of life [5]. Blakeslee (1932), a biologist who studied chromosomal mutations, succeeded in producing "synthetic new species" that conformed to predictions based on chromosomal rearrangement patterns [6]. Later, in 1974, Waclaw Szybalski popularized the term 'synthetic biology' to describe technologies that fall within the category of modern genetic engineering [7], and subsequently, Barbara Hobom used this term to describe genetically engineered bacteria created using recombinant DNA technology [8]. Over subsequent decades, the concept of synthetic biology expanded significantly as technologies advanced. In 2000, Eric Kool and other researchers defined 'synthetic biology' as a research field focusing on the synthesis of artificial organic molecules that can function within biological systems [9]. This established the core concept of synthetic biology as designing novel chemical structures not found in nature and integrating them with biological systems. On March 29, 2021, it was reported that a true living organism had been successfully synthesized through the design and assembly of genes in a laboratory environment [10]. Contemporary synthetic biology has progressed beyond simple genetic manipulation techniques into an engineering approach for designing biological systems and imbuing them with novel functions. Researchers approach this by modularizing and assembling life systems to perform specific functions. They utilize proteins with specific functions in organisms (such as DNA-binding proteins) and DNA sequences (such as sequences to which certain proteins bind) as standardized 'biological parts' that can be isolated and employed as composable biological elements [11]. In this context, this review defines synthetic biology as a field of biotechnology that involves the engineering-based design, construction, and application of biological components and systems.

This engineering approach aims to ultimately design biological systems in a manner analogous to electronic engineering, which directly connects to the concept of mass production systems, or biomanufacturing. Biomanufacturing refers to the production method that utilizes biological systems (living microorganisms, human cells, plants, animal tissues, enzymes, or *in vitro* synthetic systems) to produce commercially important value-added biological molecules [12]. This approach differs from conventional chemical engineering production methods and is widely applied across agriculture, food, energy, materials, and pharmaceutical industries. Biomanufacturing has evolved through four stages from traditional fermentation technology. The 1910s saw the beginning of first-generation biomanufacturing using single microorganisms to produce basic substances such as acetone and amino acids; in the 1940s, it developed into second-generation with using mutant microorganisms for antibiotic production; the 1980s advanced to third-generation with genetically engineered cells producing insulin, enzymes, and other biopharmaceuticals; and since the 2000s, fourth-generation biomanufacturing has emerged, combining stem cells, synthetic organisms, and artificial intelligence (AI) technologies, expanding applications to pharmaceuticals, bioenergy, artificial foods, and various other industrial fields [13]. Biomanufacturing is important in new drug development, biofuel production, environment-friendly chemical synthesis, and alternative protein production, garnering attention as a promising alternative that overcomes the limitations of conventional chemical-based manufacturing methods. Notably, biofoundries have emerged as essential infrastructure for extending the outcomes of synthetic biology research. In general, biofoundries contribute to standardizing and accelerating research processes by automating tasks such as DNA synthesis, gene assembly, and metabolic pathway optimization. While advanced biofoundries overseas often utilize such automation to scale laboratory-level results toward industrial applications, public biofoundries in Korea are more prominently characterized by their role in accelerating research and development. Thus, biofoundries play a dual role in enhancing both research efficiency and scalability. They provide a critical foundation for the integration of synthetic biology with biomanufacturing, facilitating the transition from laboratory-based technologies to industrial applications. This process can be understood as the establishment of large-scale production systems based on biotechnology, ultimately driving substantial industrial innovation.

## Scientists Looking Toward Policy: The Need to Redefine Their Role in the Era of Synthetic Biology

Synthetic biology has emerged as a transformative technology that extends beyond laboratory confines to influence industry and society at large. By enabling the design and recombination of genetic components of living organisms, it offers innovative solutions across various domains, including pharmaceutical development, biofuel production, and environmental remediation. However, the successful translation of such technological advancements into tangible societal benefit requires robust support from science and technology policy. Such policy frameworks are essential for setting R&D priorities, enhancing societal acceptance of new technologies, and managing potential risks.

The interplay between science and policy is well demonstrated by historical precedents. The Manhattan Project exemplifies how collaboration among universities, the military, industry, and government enabled science and technology to evolve beyond discovery into a powerful instrument of national policy implementation [14]. This underscores that effective utilization of science and technology requires supportive policy infrastructure. Similarly, the launch of the Soviet satellite *Sputnik* in 1957 galvanized public interest in science within the USA and catalyzed expanded governmental support for scientific research [15]. These cases illustrate that national competitiveness in advanced technologies depends on how effectively such technologies are institutionalized and disseminated through policy frameworks.

Synthetic biology presents unique policy challenges. Common metaphors such as "life as machine" or "life as text"—while useful in emphasizing utility and accessibility—have been criticized for potentially obscuring the complexity and ethical dimensions of life itself [16]. While mechanistic framings may simplify scientific understanding and facilitate technological application, they risk neglecting the intrinsic value and complexity of living systems. Since its early development in the 2000s, synthetic biology has been subject to extensive ethical and societal debate; however, growing emphasis on automation, artificial intelligence, and economic efficiency has raised concerns about a relative retreat from ethical reflection. Such shifts risk leaving the potential risks and broader societal implications of synthetic biology underexplored in policy discourse.

In this context, scientists' role extends beyond developing novel technologies within the laboratory. They must engage with the broader societal and institutional contexts in which their research is conducted and interpreted. To ensure that synthetic biology's transformative potential contributes to public benefit, a foundation for the co-evolution of technology and policy is essential, which can be achieved through collaborative governance involving scientists, policymakers, and civil society.

### Bridging Technological Advances and Governance: A Call for Co-Evolution

Synthetic biology has undergone rapid development over the past few decades, with two critical technological turning points at its core. First, the development of next-generation sequencing (NGS) technologies the mid-to-late 2000s substantially decreased DNA sequencing costs, enabling large-scale genomic analysis. In fact, based on the Human Genome as a reference, the cost of sequencing per megabase (Mb) and genome sequencing plummeted from $5,292 and $95.26 million, respectively, in 2001 to $0.008 and $689, respectively, in 2020 [17]. This cost reduction has enabled crucial data acquisition and analysis in synthetic biology, greatly expanding opportunities for gene synthesis and design. Second, with the development of gene-editing technologies such as CRISPR–Cas9 allowing precise manipulation of specific genes, the ability to design and optimize biological systems has improved dramatically. Since the discovery of the CRISPR–Cas9 system in 2012, this technology has revolutionized basic scientific research and established itself as a powerful tool for precise and efficient gene editing [18]. CRISPR–Cas9 has transcended its role as a simple research tool to become a foundational technology for innovative crop development and new therapeutic approaches, making synthetic biology increasingly sophisticated. These advancements in DNA sequencing and gene-editing technologies are becoming key drivers accelerating the development of synthetic biology, opening new possibilities in biotechnology and medicine.

While technological innovations are progressing rapidly and expectations for the future of synthetic biology are rising, the gap between the pace of technological advancement and regulatory frameworks continues to widen. Current regulatory systems cannot adequately reflect the development speed, complexity, and expanding application scope of synthetic biology products. In South Korea, the government is yet to establish clear guidelines on whether microorganisms, plants, and animals produced through synthetic biology should be classified as living modified organisms (LMOs). However, if synthetic biology is considered a genetically modified organism according to Article 2, Paragraph 2 of the "Act on Transboundary Movements of Living Modified Organisms," it would be subject to the current regulations, potentially creating significant constraints in commercialization and research development processes. When synthetic biology-based substances and products are classified as LMOs, the authorization process requires substantial time and cost. In South Korea, risk assessments take an average of 22.6 months, and the costs incurred during the review process account for approximately 26% of total LMO authorization expenses (National Assembly Committee on Industry, Trade, and SMEs, *Review Report on the Partial Amendment of the Act on Transboundary Movement of Living Modified Organisms*, September, 2022). This demonstrates that regulatory requirements can act as significant constraints on the commercialization and research and development of synthetic biology technologies. This could impede the development and innovation of synthetic biology-based technologies. In contrast, the UK is actively promoting industrial support through regulatory innovation. On October 9, 2024, the UK government announced the establishment of the Regulatory Innovation Office, designating advanced technologies such as synthetic biology as priority areas. Specifically, they stated that a key objective is to streamline regulatory procedures to enable synthetic biology-based products to reach the market safely and rapidly [19]. The United Kingdom has established a concrete example of regulatory innovation by enacting the *Genetic Technology (Precision Breeding) Act 2023* and drafting the *Genetic Technology (Precision Breeding) Regulations 2025*, thereby creating a distinct regulatory category separate from conventional LMOs. Under the current LMO regulatory framework (*e.g.*, Regulation (EC) No 1829/2003), prior authorization, safety assessments, and mandatory labeling are required. In contrast, the Precision Breeding Act applies a new regulatory framework that substantially streamlines the procedures. First, products classified under precision breeding are exempt from labeling obligations. Second, safety assessments are not mandated for species with established usage histories and long-standing consumption by significant populations. Instead, such cases undergo simplified verification and reporting procedures, through which market authorization may be granted based on prior evidence of safety. This regulatory simplification exemplifies a rational balance that reduces unnecessary procedural redundancy while maintaining safety, thereby providing an institutional foundation that promotes the commercialization of products derived from emerging technologies.

Such changes signify the establishment of a virtuous cycle structure where innovation and institutional frameworks are not viewed as separate domains but continuously interact and evolve together. While technological innovation is inherently dynamic, institutions tend to be relatively static. This inevitably creates gaps between technology and regulation, necessitating continuous learning and adjustment to reduce these disparities and enable regulation and innovation to develop concurrently. In other words, a "co-evolution" of technological innovation and legal/institutional frameworks is required [20]. For establishing synthetic biology not merely as a laboratory technology, but as an innovation impacting industry and society at large, a structure where technological development, regulation, and societal acceptance evolve simultaneously should be created. To achieve this, employing a predictive governance framework to harmoniously design the pace of technological development with regulatory systems is necessary [21].

Synthetic biology is a rapidly evolving field, expanding from laboratory experimentation to industrial applications while raising societal and ethical issues. To comprehensively understand this field, it should be analyzed from three temporal perspectives: past, present, and future. First, examining the past illuminate synthetic biology's developmental trajectory and its industrial expansion through integration with biomanufacturing. Second, surveying the present allows analysis of key issues facing synthetic biology, centered on regulatory challenges and responsible innovation. Third, envisioning the future necessitates exploring strategic responses for sustainable development of synthetic biology. To this end, we apply the anticipatory governance framework of the Organization for Economic Co-operation and Development (OECD), proposing sustainable development directions focused on five core elements: R&D promotion, strategic intelligence building, stakeholder engagement, agile regulation, and international cooperation.

Based on this analysis, this review aims to provide a balanced perspective on the technological, industrial, societal, and policy development of synthetic biology, proposing a "co-evolution" model where diverse elements interact and develop together. Specifically, as the field expands from R&D to industrial innovation, regulatory frameworks, societal acceptance, and international cooperation, we emphasize that these elements should evolve not independently but through mutual adjustment and balance. Through this approach, we seek to explore directions for the sustainable growth of synthetic biology within a system where industry, legal framework, society, and international cooperation develop in tandem, transcending mere technological innovation.

## Historical Development and National Policies

This section provides a chronological overview of the major developmental milestones and achievements in synthetic biology. It also examines the strategic policies and national initiatives that have underpinned its growth, highlighting the pivotal role of governmental support and long-term planning. By analyzing national strategies aimed at fostering synthetic biology as a critical future technology, this section seeks to contextualize current policy challenges and industrial priorities within a broad historical and geopolitical framework.

### Evolution of Synthetic Biology Technologies

**Fig. 1:**
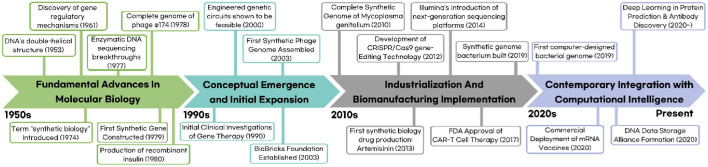
The Chronological Development of Synthetic Biology (1950s–2020s).

Synthetic biology currently lacks a universally accepted international definition [22]. However, the field has been operationally characterized through various institutional and disciplinary frameworks. The Convention on Biological Diversity (CBD) conceptualizes synthetic biology as an "extension and acceleration of modern biotechnology." The Ad Hoc Technical Expert Group (AHTEG) has proposed an operational definition describing synthetic biology as an interdisciplinary convergence of science, technology, and engineering that facilitates the understanding, design, redesign, fabrication, and modification of genetic materials, living organisms, and biological systems. Several authoritative sources have contributed complementary definitions. *Nature* characterizes synthetic biology as "the design and construction of new biological parts, devices, and systems, and the redesign of existing, natural biological systems for useful purposes." The U.S. National Human Genome Research Institute defines it as "a field of science that involves redesigning organisms for useful purposes by engineering them to have new abilities." Wikipedia offers a multidisciplinary perspective, describing it as "a multidisciplinary field of science that focuses on living systems and organisms, and it applies engineering principles to develop new biological parts, devices, and systems or to redesign existing systems found in nature" [1]. These definitional approaches collectively suggest that synthetic biology should be conceptualized not as a static construct but rather as a dynamic field that evolves concomitantly with technological advancements. It represents an expanding methodological paradigm that applies engineering principles to biological systems for translational applications. The chronological development of synthetic biology can be delineated through four distinct phases: (1) fundamental advances in molecular biology, (2) conceptual emergence and initial expansion, (3) industrialization and biomanufacturing implementation, and (4) contemporary integration with computational intelligence (Fig. 1). The initial phase (1950s–1980s) established the molecular foundations of the field. DNA double-helical structure elucidation [23] was followed by Jacob and Monod's pioneering work on molecular regulatory mechanisms and genetic control systems [24]. The 1970s witnessed the introduction of the term "synthetic biology," coinciding with methodological breakthroughs in enzymatic DNA sequencing [25] and the complete genomic characterization of bacteriophage φ174 [26]. The first synthetic gene was constructed in 1979 [27]. The 1980s marked the transition to industrial applications with recombinant insulin production [28], and the regulatory approval of mammalian cell-derived biopharmaceuticals. The second phase (1990s–2000s) encompassed the field's formalization and expansion. Initial clinical investigations of gene therapy commenced in the 1990s [29], while commercial gene synthesis was initiated in 1999 through entities such as GeneArt and Blue Heron Biotechnologies [30]. A seminal publication in 2000 demonstrated the feasibility of engineered genetic circuits [31], and the inaugural International Conference on Synthetic Biology was convened at MIT in 2004 [32]. The first synthetic phage genome was assembled in 2003 [33], concurrent with the establishment of the BioBricks Foundation, which promoted standardized biological components [34].

The third phase (2010s) was characterized by industrial implementation and biomanufacturing advancements. The complete synthetic genome of Mycoplasma genitalium was synthesized in 2010 [35]. While CRISPR/Cas9 gene-editing technology was developed [36] and plant-derived protein therapeutics were introduced in 2012 [37]. In 2013, the antimalarial compound artemisinin became the first major pharmaceutical product derived through synthetic biology [38] and Synthetic Biology 6.0 established the global research agenda [39]. Illumina introduced NGS platforms in 2014, which accelerated genomic investigations [40]. Regulatory milestones were achieved in 2017 with FDA approval of CAR-T cell therapy [40] and the initiation of *in vivo* gene-editing clinical trials [41]. By 2019, researchers had successfully constructed a functional bacterium with a completely synthetic genome [42]. The current phase (2020s) represents the convergence of synthetic biology with computational intelligence. The first computer-designed synthetic bacterial genome was completed in 2019 [43]. The COVID-19 pandemic accelerated the commercial deployment of mRNA vaccines in 2020 [44], demonstrating synthetic biology's capacity for rapid response to global health challenges. The formation of the DNA Data Storage Alliance in 2020 reflected growing interest in nucleic acid-based data storage technologies [45]. Recent advancements include the application of deep learning algorithms to protein structural prediction [46] and antibody discovery [47], indicating the accelerated integration of AI with biological engineering.

In conclusion, synthetic biology has evolved from its foundations in molecular biology through industrial implementation to its current AI-augmented innovation phase. The emerging paradigm integrates biomanufacturing capabilities, computational intelligence, and digital technologies, thereby shaping the trajectory of next-generation biotechnology.

### National Strategic Policies: US, UK, Japan, and Korea

The rapid advancement of synthetic biology can largely be attributed to strategic policies and long-term national planning implemented by individual countries. Owing to synthetic biology's broad impact across science, technology, and industry, leading nations such as the USA and the UK have established systematic roadmaps to position themselves at the forefront of R&D. In response, follower countries like Japan and South Korea have adopted tailored national strategies to enhance competitiveness in the global bioeconomy. Within this policy landscape, technology roadmaps serve as not only tools for forecasting future market demand, but also strategic guides for setting R&D priorities, developing target products and technologies, and informing policy design [48].

The USA has designated synthetic biology as a foundational technology for its next-generation bioeconomy, developing a comprehensive strategic framework detailed in "Engineering Biology: A Research Roadmap for the Next-Generation Bioeconomy" [49]. This document delineates a national approach to systematically advance synthetic biology capabilities and facilitate their cross-sectoral integration to support economic development and technological sovereignty. The USA roadmap is structured around four fundamental technical domains: genome engineering and synthesis, biomolecular and cellular engineering, host and consortia engineering, and data integration and automation. These core technological platforms are applied across five priority sectors: industrial biotechnology, health and medicine, food and agriculture, environmental biotechnology, and energy. Specific strategic objectives include the development of sustainable production methodologies for chemicals, fuels, and advanced materials in the industrial sector; advancement of gene therapies and personalized medicine in healthcare; enhancement of agricultural productivity and climate resilience in food systems; implementation of ecosystem restoration and pollution remediation in environmental applications; and development of next-generation biofuels to advance carbon neutrality objectives [50].

The UK has similarly published a national synthetic biology framework, "A Synthetic Biology Roadmap for the UK" (2012), positioning this technology as a catalyst for economic growth and sustainable innovation [51]. The UK strategy aims to establish a globally competitive synthetic biology sector through several prioritized initiatives. Primarily, it focuses on strengthening foundational scientific and engineering capabilities, particularly in DNA synthesis technologies, genetic manipulation methodologies, and metabolic engineering approaches, while concurrently investing in research infrastructure and human capital development. Secondarily, the roadmap emphasizes on responsible research and innovation (RRI) principles, integrating ethical considerations and regulatory frameworks into research processes and fostering public engagement to enhance societal acceptance. Additionally, it supports commercialization pathways and industrial applications by facilitating technology transfer mechanisms and providing targeted support for startups and small-to-medium enterprises (SMEs) to translate research outcomes into commercially viable products. The framework promotes collaborative relationships between academic institutions and industry to accelerate the application of synthetic biology across sectors including medicine, energy, agriculture, and environmental management. Finally, the UK strategy aims to establish the nation as a global nexus for synthetic biology through international collaborative networks and leadership in global standardization and policy development, particularly through coordinated engagement with the USA, European Union, and China [52].

Japan has integrated synthetic biology into its national policy framework [53], though it has not published a standalone strategic roadmap comparable to those of the USA and UK. Instead, Japan conceptualizes synthetic biology as a key enabling technology within its comprehensive bioeconomy strategy. Rather than emphasizing technological advancement in isolation, Japan's approach focuses on the application of synthetic biology to enhance biomanufacturing capabilities, sustainable agricultural practices, and biopharmaceutical innovation. The country is strategically leveraging its established expertise in fermentation technologies and cell-based methodologies, integrating them with synthetic biology approaches to drive industrial applications and enhance competitive positioning. Japan is also prioritizing the reinforcement of domestic biomanufacturing supply chains to ensure economic security, while actively incorporating AI and data-driven design methodologies to optimize the efficiency of synthetic biology applications. This strategic approach reflects a calculated effort to consolidate national technological capabilities while adapting to evolving global bioeconomic trends.

South Korea has articulated a national synthetic biology strategy [54] with quantifiable objectives, aiming to achieve 90% technological parity with the USA in synthetic biology capabilities by 2030. The Korean approach encompasses four principal strategic initiatives: development of core technological platforms, implementation of targeted flagship projects, establishment of biofoundry infrastructure, and expansion of international collaboration networks and talent development programs First, six foundational technologies—biomolecular design, DNA/RNA synthesis, biosystem construction, automation, and scale-up methodologies—have been designated as priority areas, with phased R&D support extending from fundamental research to commercial applications. Second, nine flagship projects spanning healthcare, environmental sustainability, and materials science have been initiated to address global challenges, including AI-augmented antibody design, greenhouse gas conversion processes, plastic-degrading microorganism development, and photosynthetic efficiency enhancement. Third, substantial investments are being directed toward biofoundry infrastructure to catalyze innovation in biomanufacturing capabilities. A centralized national biofoundry will be established and subsequently expanded into sector-specific facilities, aiming to complete a comprehensive national biomanufacturing innovation platform by 2030. Finally, South Korea is intensifying global collaborative networks and developing interdisciplinary expertise across biology, AI, and engineering disciplines. Through this integrated strategic approach, the nation aims to cultivate emerging industries based on synthetic biology applications, enhance global competitive positioning, and establish the foundations for a sustainable, bio-based economy centered on advanced biomanufacturing methodologies.

Heterogeneity in national regulatory frameworks exerts significant influence on innovation trajectories and industrial development in the synthetic biology sector. While nations such as the USA and the UK have implemented regulatory reforms to accommodate technological advancement, South Korea continues to regulate synthetic biology applications within the established framework for LMOs. This regulatory paradigm potentially constrains commercialization pathways and industrial implementation of synthetic biology technologies. However, mere adherence to existing regulatory structures is insufficient to ensure comprehensive biosafety and an adaptive regulatory architecture that effectively balances technological innovation imperatives with societal trust considerations is required. Owing to the inherent dual-use potential and biosecurity implications associated with synthetic biology, the institutionalization of RRI principles is essential. In this context, we apply the OECD framework for anticipatory governance to analyze policy interventions and strategic approaches that can secure sustainable development pathways in synthetic biology.

Synthetic biology has emerged as a pivotal strategic technology driving industrial innovation and enhancing national competitiveness metrics. Recognizing this strategic significance, numerous countries are implementing multidimensional approaches to support synthetic biology development extending beyond traditional R&D investments to encompass biomanufacturing advancement, regulatory system modernization, and expanded international collaborative networks. The USA and the UK have formulated discrete roadmaps that establish coherent pathways connecting fundamental research to industrial applications. Concurrently, Japan and South Korea have integrated synthetic biology within comprehensive national bioeconomy frameworks, emphasizing biomanufacturing innovation and supply chain resilience. Notably, South Korea is accelerating commercialization processes and enhancing global competitive positioning through strategic investments in biofoundry infrastructure and expanded international partnerships.

Despite these strategic initiatives, regulatory challenges and societal acceptance considerations remain significant impediments in the translational pathway from innovation to industrial application. Existing regulatory architectures frequently demonstrate insufficient adaptability to accommodate the accelerated pace of technological advancement. As novel technologies emerge, ensuring their compliance with safety and ethical standards while simultaneously securing societal approval through inclusive dialogic processes becomes increasingly critical.

Therefore, the following section examines the regulatory challenges and societal acceptance dimensions associated with biomanufacturing industrialization, and investigates policy directives to address these issues effectively.

## Current Challenges

One of the most pressing issues in the field of synthetic biology is the regulatory framework. Particularly, divergent interpretations of LMOs and gene-edited organisms (GEOs) have resulted in varying regulatory approaches across countries, significantly affecting technological innovation and industrialization. While some countries continue to apply existing legislation under the framework of the LMOs Act, others have established separate criteria to promote innovation. These discrepancies have widened gaps in R&D as well as market formation across national borders.

Moreover, owing to the dual-use nature of synthetic biology—where technologies may be applied for both beneficial and potentially harmful purposes—the importance of biosafety and RRI is increasingly emphasized.

### Conflicts and Gaps in Existing Regulatory Frameworks

As the development of synthetic biology-based products gains momentum and accelerates commercialization, active discussions regarding whether these products should be regulated under existing biotechnology frameworks or new regulatory systems should be established are underway. Concerns have been raised that current regulatory regimes may not sufficiently reflect the rapid pace of technological advancement, prompting some countries to introduce or consider customized regulatory approaches tailored to the characteristics of synthetic biology products [55].

In the USA, regulatory policy is grounded in scientific evidence and follows a risk-based approach. Rather than regulating based on the development process or technological method, the USA system focuses on the characteristics and potential risks of the final product itself [56]. In 2022, President Biden issued Executive Order 14081, *Advancing Biotechnology and Biomanufacturing Innovation for a Sustainable, Safe, and Secure Bioeconomy*, aimed at accelerating innovation in biotechnology and biomanufacturing. In response, the USDA, FDA, and EPA released the "Plan for Regulatory Reform under the Coordinated Framework for the Regulation of Biotechnology," which outlines reform strategies for major biotechnology products, including genetically modified plants, animals, microorganisms, human therapeutics, and biologics. This plan aims to enhance regulatory clarity and efficiency, reduce redundant oversight, foster a transparent and predictable regulatory environment, maintain consistency across agencies, and support technological innovation [52]. The USA approach serves as a strategic model for harmonizing safety and industrialization of synthetic biology and could provide a valuable reference for other countries designing their regulatory systems.

The UK has also transitioned away from a one-size-fits-all regulatory model and is adopting a scientific and proportionate approach to LMOs governance [57]. The cornerstone of this transformation is the *Precision Breeding Act*, enacted in March 2023. Although the UK is a party to the Cartagena Protocol on Biosafety, which generally requires organisms modified by gene-editing technologies to be classified as LMOs, the UK government has determined that organisms produced through techniques defined in the Precision Breeding Act differ from those outlined in the Protocol's definition of modern biotechnology. Article 3 of the Cartagena Protocol defines a LMO as “any living organism that possesses a novel combination of genetic material obtained through the use of modern biotechnology.” However, the definition of LMOs under the Protocol has primarily focused on organisms modified through the insertion or deletion of foreign genes. In contrast, organisms produced through precision breeding often exhibit alterations that are difficult to distinguish from naturally occurring mutations [58]. On the basis of this distinction, the UK introduced a new legal framework that excludes certain GEOs from traditional LMOs regulations and places them in a regulatory category aligned with conventional breeding. Ultimately, the key issue lies in determining the conditions under which a case may be regarded as “precision breeding.” Only when classified as precision breeding can an organism be exempted from the application of the *Regulation (EC) No 1829/2003* on genetically modified organisms. The UK *Genetic Technology (Precision Breeding) Act 2023* specifies four criteria for this classification. First, all genomic features of the organism must result from the application of modern biotechnology. Second, these features must be stably inheritable across successive generations. Third, the genomic characteristics that arise must, in principle, be achievable through conventional breeding processes. Fourth, features resulting from artificial modification techniques other than modern biotechnology are not to be included. Meanwhile, in July 2023, the European Union (EU) proposed the *New Genomic Techniques (NGT) Plant Act*. This legislation is characterized by its differentiated regulatory approach depending on the type of technology. NGT1 technologies, which involve modifications without the introduction of foreign genes, are exempt from existing LMO regulations such as risk assessments. By contrast, NGT2 and NGT3 technologies remain subject to the current LMO framework or are managed through simplified procedures [59]. Thus, while both the UK and the EU share a common orientation toward regulatory streamlining, their approaches diverge. The UK adopts a single category of “precision breeding” under which a new, relaxed regulatory framework is applied, whereas the EU implements a differentiated approach by applying distinct regulatory measures according to the type of NGT (NGT1, NGT2, NGT3).

In contrast, South Korea currently classifies synthetic biology technologies under the umbrella of modern biotechnology, which means they are likely to be regulated as LMOs [50]. Consequently, R&D activities involving synthetic biology are subject to the *Act on Transboundary Movements of Living Modified Organisms*. For example, researchers developing new microorganisms must obtain approval from the Korea Disease Control and Prevention Agency, as stipulated in Article 22-2 of the Act, which imposes a considerable administrative burden. Furthermore, even non-pathogenic microorganisms lacking a specified species name are subject to risk assessment by designated regulatory authorities (Article 7-2). In a biofoundry, numerous experiments and iterative Design–Build–Test–Learn (DBTL) cycles are rapidly executed within a single loop. However, if approval from relevant authorities is required at each stage under the current *Living Modified Organism Act*, the speed and efficiency of these loops would be severely hampered. Consequently, such requirements may function as a structural constraint that prevents biofoundries from fully realizing their innovative potential and transformative impact. Unlike the regulatory reforms seen in the USA and the UK, South Korea continues to manage synthetic biology technologies within the conventional LMOs regulatory framework, which may serve as a significant barrier to both research and commercialization efforts.

### RRI

The rapid advancement and commercialization of synthetic biology have brought not only regulatory challenges, but also the critical need for RRI. Just as differences in regulating LMOs and gene-editing technologies can significantly impact innovation, RRI plays a pivotal role in balancing technological advancement with public acceptance. RRI is not limited to compliance with legal regulations but represents a comprehensive approach that integrates social responsibility and ethical considerations into every stage of research and innovation. Its aim is to foster the participation of diverse stakeholders—including scientists, policymakers, industry, and civil society—in order to deliberate on the potential benefits and risks of emerging technologies in advance, thereby guiding technological development in alignment with societal values.

The core principles of RRI can be summarized in four dimensions. First, Inclusiveness ensures that diverse members of society are engaged in the research process so that technological development contributes not only to the interests of a few but to the benefit of society as a whole. Second, Anticipation and Responsiveness highlight the importance of predicting the potential impacts of technology and maintaining flexibility in addressing unforeseen social and ethical concerns. Third, Openness and Transparency promote public trust and constructive debate by ensuring transparent disclosure of research processes and outcomes. Finally, Reflexivity encourages researchers to continuously reflect on the broader societal implications of their work [47]. Synthetic biology, particularly, poses unique concerns related to biosecurity and dual-use, necessitating a cautious and ethical approach to R&D. An illustrative example of such concerns was presented in the December 2024 issue of *Science*, which published a paper titled "*Confronting Risks of Mirror Life.*" The article analyzed the potential risks associated with the experimental creation of mirror life—synthetic organisms with chirality opposite to that of natural biological systems. Since these entities do not exist in nature, they may evade immune detection, disrupt ecosystems, and produce unpredictable biological effects [60]. While research into mirror life may arise from scientific curiosity, neglecting its societal and ethical implications could lead to severe and unforeseen consequences.

In response to such risks, the UK government has released new user and provider guidelines on synthetic nucleic acids on October 8, 2024, which aim to strengthen biosecurity and enhance research accountability. RRI primarily involves the principle of maintaining a balance between technological progress and societal trust. The UK guidance reflects this principle by instituting mandatory customer and sequence screening procedures to prevent misuse, promote transparency in research practices, and ensure compliance with international export control regulations [61].

## Future Outlook

**Fig. 2:**
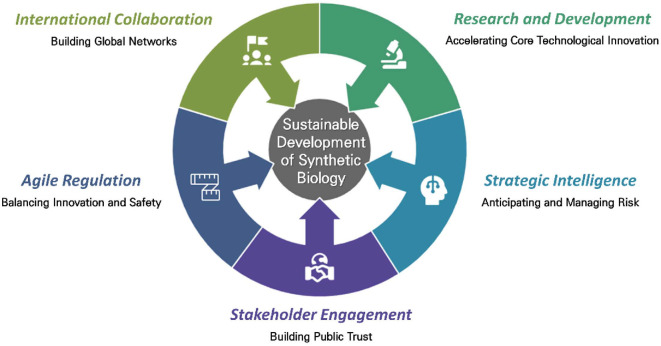
The Future of Synthetic Biology: Strategic Response Plans for Sustainable Development.

Synthetic biology is emerging as a technological innovation and a transformative field with broad implications for industry and society. As developments such as the expansion of biomanufacturing, convergence with AI, and efforts toward global regulatory harmonization continue to unfold, a strategic approach to the development and application of this technology is essential. Particularly, the OECD has proposed a framework for anticipatory governance of emerging technologies, offering policy guidance for the responsible development and societal acceptance of advanced technologies like synthetic biology [17]. Accordingly, this review explores the future strategies for sustainable and responsible advancement of synthetic biology, focusing on five key areas emphasized by the OECD framework: R&D promotion, strategic intelligence, public trust-building, agile regulation, and international cooperation (Fig. 2).

### R&D: Accelerating Core Technological Innovation

To achieve the successful industrialization of synthetic biology, the advancement of biofoundries and biomanufacturing is essential. Biofoundries serve as foundational infrastructure that accelerates the transition from R&D to commercial-scale production, facilitating the practical application of synthetic biology technologies. Since the launch of the Global Biofoundry Alliance (GBA) in Kobe, Japan, in 2019, global interest in biofoundries has significantly increased [62]. These platforms enhance research efficiency through automation, reduce the risk of experimental failure, and enable rapid process optimization. However, challenges such as rapid technological obsolescence and high initial investment costs remain [63]. Therefore, biofoundries must evolve beyond basic automation facilities to function as knowledge hubs that link research with industry and bridge fundamental and applied research, thereby supporting the effective industrial transition of synthetic biology. For successfully integrating biofoundries into biomanufacturing, three core factors are required: scale, demand, and strain optimization [64]. First, scalable production must be designed to achieve cost-effectiveness, leveraging AI and automation for optimized processes. Second, sustainable biomanufacturing requires a stable market and long-term demand secured through offtake agreements with governments and major corporations to build market confidence. Third, the acquisition and optimization of high-yield microbial strains—enabled by gene editing, AI-driven strain engineering, and synthetic biology platforms—are critical to manufacturing success. To realize these goals, standardized biofoundry design and optimal production scale must be established. Additionally, adopting AI-driven and automated production models, building cost-effective mass production systems, and aligning with global standards are central strategies for advancing biomanufacturing. Through such efforts, biomanufacturing can become a practical foundation for translating synthetic biology research into industrial applications.

### Strategic Intelligence: Anticipating and Managing Risk

Synthetic biology, as a convergence of biology, technology, and AI, represents a typical "black box" field, where the interactions among technologies are difficult to predict. Thus, establishing a strategic intelligence system that enables continuous monitoring and assessment of technological impacts is crucial for risk management. The field is currently advancing through iterative DBTL cycles, with key enabling technologies including genome editing, synthetic DNA, and artificial cell construction [65]. These technologies are rapidly evolving, necessitating ongoing assessments of their societal and environmental impacts to minimize foreseeable risks. Recognizing this need, the South Korean National Assembly passed the "Synthetic Biology Promotion Act" on March 11, 2025, during the 423rd plenary session of the Science, ICT, Broadcasting and Communications Committee [66]. The Act includes guidelines for R&D (Article 27) and mandates the implementation of a risk management and safety assessment system (Article 28), reflecting a governmental commitment to proactively forecast and manage the risks associated with synthetic biology. Notably, generative AI development enables refined simulations and predictive models for assessing biosecurity threats and evaluating the potential societal and ethical implications of emerging technologies. These capabilities signify a shift toward a structured and anticipatory approach to managing the risks of synthetic biology.

### Stakeholder Engagement: Building Public Trust

Establishing societal trust is as essential as technological innovation for realizing a full-fledged bioeconomy. McKinsey has projected that up to 60% of physical inputs in future manufacturing could be bio-based, driven by biotechnology applications [67]. To achieve this vision, engagement from a wide range of stakeholders—including policymakers, academia, private enterprises, non-profit organizations, labor unions, and civil society—is indispensable. A frequently cited historical precedent underscoring the importance of societal discourse is the Asilomar Moratorium of the 1970s. At that time, concerns about the safety of recombinant DNA technology led scientists to voluntarily suspend research and initiate public discussions that produced ethical guidelines, enabling genetic research to advance under sustained public trust [68].

However, more recent scholarship has challenged the view of Asilomar as an unqualified success of scientific self-regulation. Hurlbut (2015) argues that the 1975 conference adopted a technology-centric, elite-driven approach, focusing narrowly on the technical dimensions of risk while neglecting ecological, ethical, and societal issues. By granting scientists authority to define the scope of risk and regulation, the process, he contends, relegated the public and legal systems to passive roles, thereby undermining democratic participation and accountability [69]. Thus, Asilomar should be remembered not only as a landmark in scientific governance but also as a case illustrating the limitations of technocratic, self-regulatory approaches.

Contemporary policy practices highlight ongoing efforts to address these limitations. In the United Kingdom, real-time online platforms capture public sentiment toward LMOs, enabling the government to integrate public opinion into policy agendas [70]. In Japan, an online community allows consumers to share experiences of cultivating and tasting GABA-enriched tomatoes developed through synthetic biology [71]. South Korea has also sought to strengthen societal dialogue: during the drafting of the "Synthetic Biology Promotion Act," the government conducted a public survey to gather input on whether the legislation should prioritize promotion or regulation [72]. These initiatives represent meaningful attempts to incorporate stakeholder perspectives into science and technology policymaking.

Looking ahead, systematic and continuous channels of communication will be necessary. Ultimately, scientists, policymakers, and citizens must develop a shared understanding of the values and directions of synthetic biology, supported by bi-directional communication. Governments and research institutions should clearly articulate both scientific facts and the broader societal implications of emerging technologies, while citizens must be empowered to participate actively in policymaking. Such mutual trust is indispensable for ensuring the stable and sustainable development of synthetic biology.

### Agile Regulation: Balancing Innovation and Safety

As an emerging and still-evolving technology, synthetic biology faces a high risk of regulatory overreach that may hinder innovation if uniform, rigid regulatory frameworks are applied before institutional maturity is achieved. Regulatory systems that rely solely on the current characteristics of the technology may fail to accommodate its future potential. Therefore, instead of relying strictly on the precautionary principle, a more flexible and prudent regulatory approach is required. This means adopting *prudent vigilance*, which allows for R&D to proceed even in the absence of complete scientific evidence, while concurrently strengthening the evidence base through continuous risk assessment [73].

To effectively support the development of synthetic biology and manage associated risks, a balance between *standards* and *regulations* should be achieved. When new technologies emerge, existing legal frameworks are often insufficient to provide appropriate oversight. In such cases, early-stage governance should be guided by technical standards and guidelines, with a gradual transition toward formal regulatory systems. This calls for an *adaptive governance* approach that evolves in tandem with scientific and technological advances [74]. By leveraging the data accumulated during R&D activities, soft-law instruments such as voluntary standards and technical guidance can be established, which can later inform the design of formal regulatory structures. This progressive mechanism not only facilitates technological innovation, but also helps build public trust and promote a responsible research environment.

### International Collaboration: Building Global Networks

Synthetic biology is a multidisciplinary technology with the potential to address global challenges such as climate change, food security, and public health, thereby underscoring the necessity of international collaboration beyond the interests of individual nations. However, as synthetic biology emerges as a strategic technology, intensified competition among countries has further highlighted the importance of establishing institutional foundations for trust-building and joint research. Among international cooperation platforms, the International Genetically Engineered Machine (iGEM) competition provides a particularly noteworthy example. Initially designed for undergraduate and graduate students, iGEM has since expanded into a global framework that also includes high school students, entrepreneurs, and community laboratories. More than a technical competition, iGEM integrates Human Practices, enabling participants to directly engage with the principles of Responsible RRI. Through this experience, participants not only strengthen their technical expertise but also cultivate ethical reflection, social engagement, and capacities for responsible innovation. Moreover, iGEM has frequently served as a springboard for expanded collaborative research and entrepreneurial initiatives. Companies such as LabGenius and Puraffinity (formerly CustoMem), both founded by iGEM alumni, exemplify how an educational platform can serve as a conduit for industrial innovation [75]. While iGEM plays a crucial role in education and early-stage innovation, the GBA illustrates another approach to promoting international collaboration by focusing on research infrastructure and institutional frameworks. The GBA establishes transnational infrastructures for biofoundry operations and promotes open science and interoperability by facilitating the sharing of protocols, resources, and standards across borders. This institutional emphasis complements iGEM’s focus on education and the dissemination of RRI. Overall, iGEM and the GBA represent distinct yet complementary pathways for fostering global collaboration. iGEM emphasizes the training of next-generation researchers, inclusiveness, and the practice of responsible innovation, whereas the GBA provides a long-term foundation for cooperation through institutional and infrastructural capacity building. Expanding and strengthening such multi-layered approaches will be essential for building a sustainable, cooperative, and RRI-oriented global ecosystem for synthetic biology.

## Conclusion

Synthetic biology is forming a new paradigm that transforms not only laboratories, but also industry and society at large. With the convergence of biomanufacturing and AI, the pace of innovation continues to accelerate, playing crucial roles in diverse fields including drug development, sustainable energy, food security, and environmental problem-solving. However, the reality that legal and institutional frameworks and societal acceptance cannot keep pace with technological advancement remains an important challenge to be addressed. In this review, we examined the developmental trajectory of synthetic biology and explored directions for sustainable development by applying the predictive governance framework given by the OECD. Particularly, based on a 'co-evolution' model where technology and legal/institutional frameworks develop harmoniously together, we proposed sustainable growth strategies centered around five key elements: R&D promotion, strategic intelligence building, stakeholder engagement, agile regulation, and international cooperation. Synthetic biology represents a field where rapidly advancing technological characteristics and regulatory responses influence and evolve with one another. Therefore, the regulations should adapt gradually and support technological development without conflicting with technological innovation. This is not simply about permitting technological innovation, but is an essential approach to securing safety and societal trust during industrialization. To achieve this, a balance point that maintains prudent vigilance without hindering technological innovation must be found by introducing standards and data-driven agile regulations. Legal and institutional frameworks should be designed to not merely control technology, but evolve alongside technology and harmonize with industry and society. Additionally, global collaboration platforms such as iGEM can serve as important means to reduce technological gaps between countries and realize the democratization of science and technology. As synthetic biology transitions from R&D to industrial innovation, regulatory harmonization and research guideline development through international cooperation become essential, and this can be a part of legal and institutional co-evolution. The development of synthetic biology must occur within a process where technology and regulation interact and change together, requiring flexible regulation and cooperative governance. Future developmental directions depend on our choices and preparation, and to lead the new paradigm of bio, academia, industry, government, and civil society must collaborate closely to maintain continuous discussion and strategic response.
